# Implementation of the Acute Inpatient Medicine—High Reliability, Learning Environment, and Workforce Development Initiative (AIM‐HI) in rural Veterans Health Administration hospitals: A mixed methods evaluation protocol

**DOI:** 10.1002/jhm.13474

**Published:** 2024-08-16

**Authors:** Heather M. Gilmartin, Brigid Connelly, Marguerite Daus, Edward Hess, Chelsea Leonard, Brianne Morgan, John P. Nolan, Paige Perry, Heidi Sjoberg, Soumya Subramaniam, Melver L. Anderson

**Affiliations:** ^1^ Denver/Seattle Center of Innovation for Veteran‐Centered and Value Driven Care, VA Eastern Colorado Healthcare System Aurora Colorado USA; ^2^ Department of Health Systems, Management and Policy Colorado School of Public Health, University of Colorado, Anschutz Medical Campus Aurora Colorado USA; ^3^ Independent Researcher, Veteran Partner El Dorado Arkansas USA; ^4^ VHA Hospital Medicine, Specialty Care Program Office, Veterans Health Administration Washington District of Columbia USA; ^5^ VA Eastern Colorado Healthcare System Hospital Medicine Section Aurora Colorado USA; ^6^ Division of Hospital Medicine University of Colorado Anschutz School of Medicine Aurora Colorado USA

## Abstract

**Introduction:**

Few rural hospital medicine programs include workforce development training that provides social and professional support for interdisciplinary teams. Even fewer include training that creates supportive learning environments that result in higher staff satisfaction, lower burnout, and reduced turnover. The Acute Inpatient Medicine—High Reliability, Learning Environment, and Workforce Development Initiative (AIM‐HI) aims to create supportive learning environments in Veterans Health Administration (VA) rural hospital medicine teams.

**Methods:**

AIM‐HI is a type II hybrid implementation study utilizing a convergent mixed methods approach to evaluate the Relational Playbook, a workforce development intervention, and three implementation strategies: behavioral nudges, learning and leadership collaboratives, and leadership coaching. AIM‐HI implementation will occur in waves, enrolling additional hospitals every 12 months. In the first wave, AIM‐HI will be implemented at three tertiary VA hospitals that treat at least 1000 rural Veterans annually and have an active inpatient hospital medicine program. The primary outcomes in year 1 will be the acceptability, appropriateness, and feasibility of AIM‐HI assessed through participant surveys and interviews. In subsequent years, trends in the learning environment, job satisfaction, burnout, and turnover scores will be assessed using a linear mixed‐effect model.

**Discussion:**

The anticipated impact of AIM‐HI is to evaluate the utility of the implementation strategies and assess trends in Playbook intervention outcomes. The Playbook has strong face validity; however, before large‐scale adoption across the VA enterprise, it is essential to establish the acceptability, appropriateness, and feasibility of the Playbook and implementation strategies, as well as to gather data on AIM‐HI effectiveness.

## INTRODUCTION

The field of hospital medicine is critical to effective functioning of healthcare systems, ensuring patients receive high‐quality care during their hospital stays and helping to drive improvements in patient outcomes and healthcare delivery.^1^ In the Veterans Health Administration (VA), hospitalists provide direct inpatient care to over 870,000 inpatients annually.^2^ They supervise residents, collaborate care with nurse practitioners (NPs) and physician assistants (PAs), perform bedside procedures, conduct perioperative consultation, and are involved with various hospital committees and leadership roles.^1^ Hospitalists have a deep understanding of the inner workings of inpatient care and maximize the capabilities of interdisciplinary healthcare teams by building relationships that foster information sharing and efficient care coordination.^1^

There are over 50,000 practicing hospitalists in the United States.^1^ The well‐being of the hospitalist workforce has been an increasing concern due to negative impacts of the COVID‐19 pandemic,^3^ along with increasingly complex patients, hospital systems, and unpredictable and often high workloads.^4^ In rural communities, the number of physicians per capita is significantly lower compared to non‐rural communities.^5^ There are higher rates of burnout^6^ and perpetual staffing shortages.^4^ Retaining the current hospitalist workforce is critical for maintaining the viability of rural hospitals and ensuring prosperity of these communities.^5^ Various professional issues have been noted to negatively influence rural physician and nurse retention, including social and professional isolation and a lack of continuous education and professional development opportunities.^4^, ^7^ Evidence suggests workforce development interventions that target social and professional support for interdisciplinary teams, while respecting local context, can create supportive learning environments that result in higher satisfaction, lower burnout and turnover.^8^ However, these interventions are not routinely available in rural healthcare settings. The Acute Inpatient Medicine—High Reliability, Learning Environment, and Workforce Development Initiative (AIM‐HI) was developed to bring the Relational Playbook (henceforth Playbook), an evidence‐based workforce development intervention,^9^ to rural VA inpatient hospital medicine physicians, nurses, NPs, PAs, social workers, and pharmacist clinical leaders.

The goals of AIM‐HI are to improve learning, teamwork, and high reliability among rural inpatient hospital medicine teams by fostering supportive learning environments. We hypothesize that teams consistently implementing the Playbook practices will experience increased satisfaction and safety climate, as well as reduced burnout and turnover compared to current conditions.^8^ Implementation of the Playbook is guided by the Relational Playbook Model of Change,^9^ blended with the core elements of the rapid learning health system (LHS) model.^10^ The rapid LHS model is designed to spur transformation by leveraging existing data sources and infrastructure to access and apply evidence in real time. It draws knowledge from real‐world processes to promote innovation adoption and system change, based on rigorous quality improvement or research methods.^10^ Preimplementation contextual assessment, adaptations, and program evaluation are guided by the Practical, Robust Implementation and Sustainability Model (PRISM).^11^ This work will address a gap in the literature on integrating workforce development interventions with LHS concepts. It will test the utility of the Relational Playbook Model of Change to guide AIM‐HI scale‐up and sustainment efforts. Finally, it will provide evidence on the feasibility and effectiveness trends of a hospital medicine, team‐focused, workforce well‐being program.

## AIM‐HI METHODS

### Description of Relational Playbook intervention

The Playbook is a workforce development intervention implemented by clinical leaders in the inpatient medicine setting. The Playbook is based on research conducted in VA cardiac catheterization laboratories that identified concepts and practices to cultivate and sustain supportive learning environments.^8^, ^9^, ^12^ Learning environments are the educational approaches, cultural context, and settings in which teaching and learning happens.^13^ Supportive learning environments empower teams to trial, adapt, and rapidly adopt innovations while using highly reliable work practices (e.g., checklists) to ensure patient safety.^14^, ^15^

The Playbook concepts are grounded in the fields of positive psychology, team science, servant leadership, the VA Whole Health, and Clinical Team Training models. These five concepts informed the development of the Playbook chapters: (1) creating a positive culture; (2) teamwork; (3) leading teams; (4) creating joy in work; and (5) communication and high reliability.^8^, ^9^, ^12^ The Playbook chapters include education and 50 evidence‐based interventions. Roll‐out of the Playbook spans 5 months, with a new Playbook chapter assigned each month. Participants complete brief asynchronous educational modules weekly and then select and implement specific Playbook interventions into team meetings, trainings, or one‐on‐one interactions. An engaged leader integrates these practices into the daily work of a team, aiming to transform relationships, communication, and work structures. The AIM‐HI program requires approximately 30 min of self‐study each week. The time needed to implement different practices varies from 5 to 60 min weekly.

The Playbook was piloted in a single VA cardiac catheterization laboratory by a highly engaged nurse manager. Based on the success of the pilot, AIM‐HI was developed to translate the Playbook practices to rural hospital medicine teams. AIM‐HI is funded by the VA Office of Rural Health with the support of the VA National Hospital Medicine Program and VA Office of Nursing Services. The study has been approved by the Colorado Multiple Institutional Review Board as a quality improvement program, exempt human subjects research.

### Intervention and study design

**Figure 1:**
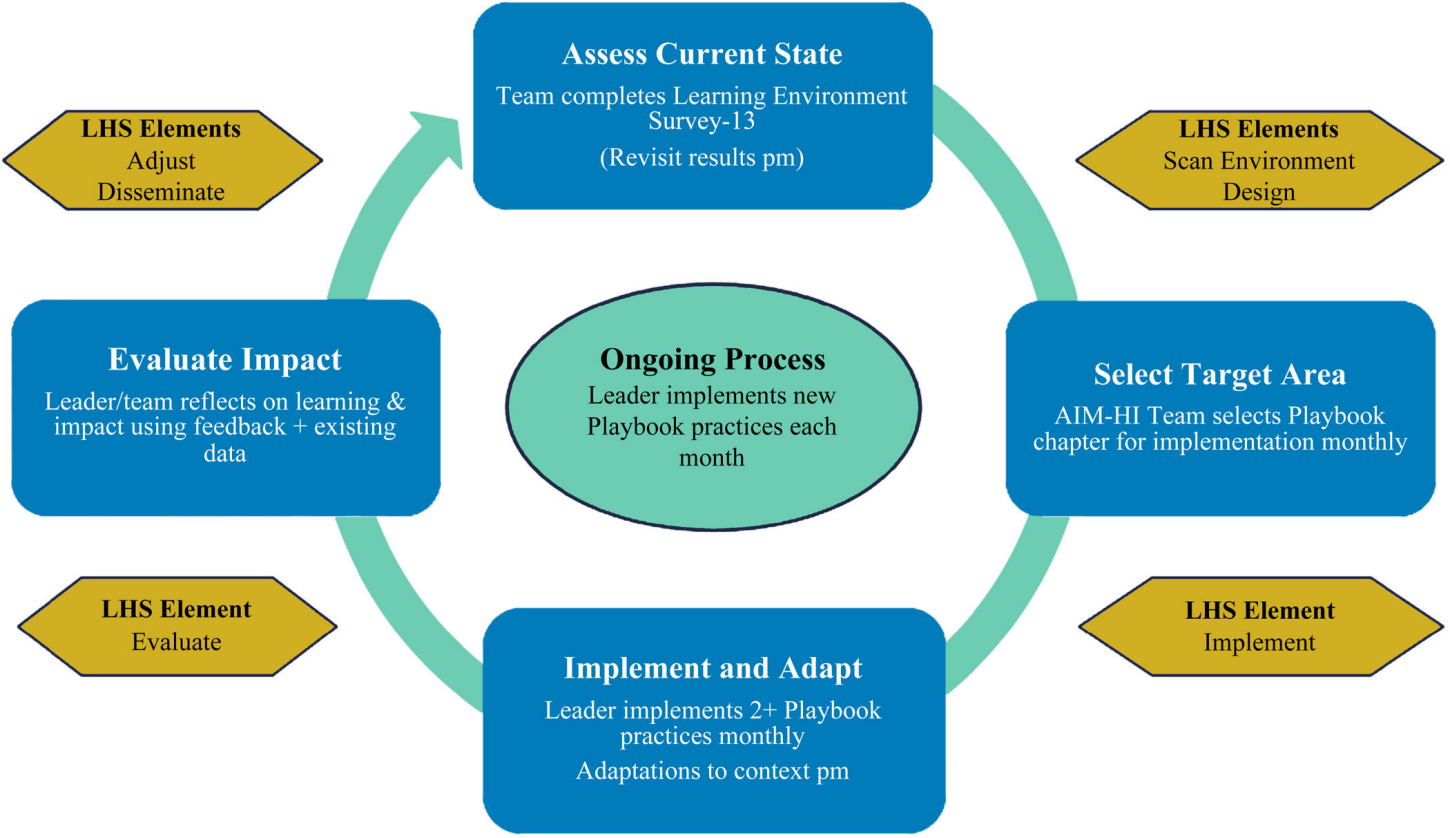
Relational Playbook model of change guided by rapid learning health system model illustrates the iterative, data‐driven learning health system approach utilized by the Acute Inpatient Medicine—High Reliability, Learning Environment, and Workforce Development Initiative participants to implement and evaluate their efforts. Participants are guided through a process of *assessing the current state* of their hospital medicine team's learning environment, *selecting target areas* for improvement, *implementing and adapting* selected Playbook practices to fit their team context, then *evaluating impact*. The process of assessment, selection, implementation, and evaluation continues iteratively throughout the 5‐month implementation phase. LHS, learning health system.

We will use the iterative and data‐driven approach of the Relational Playbook Model of Change,^9^ blended with the core elements of the rapid LHS model^10^ to guide the implementation and evaluation of AIM‐HI (Figure 1). The Relational Playbook model was developed to enable healthcare teams to continually learn, adapt, and innovate to create supportive learning environments. We will assess the Relational Playbook Model domains using convergent mixed methods, collecting facility, and team‐level data. The quantitative and qualitative data will be analyzed separately, then compared to see if the quantitative and qualitative data confirm or refute each other.^16^ The Relational Playbook Model will be utilized in each phase of our intervention: preimplementation, implementation, evaluation, and sustainment.^17^

**Figure 2:**
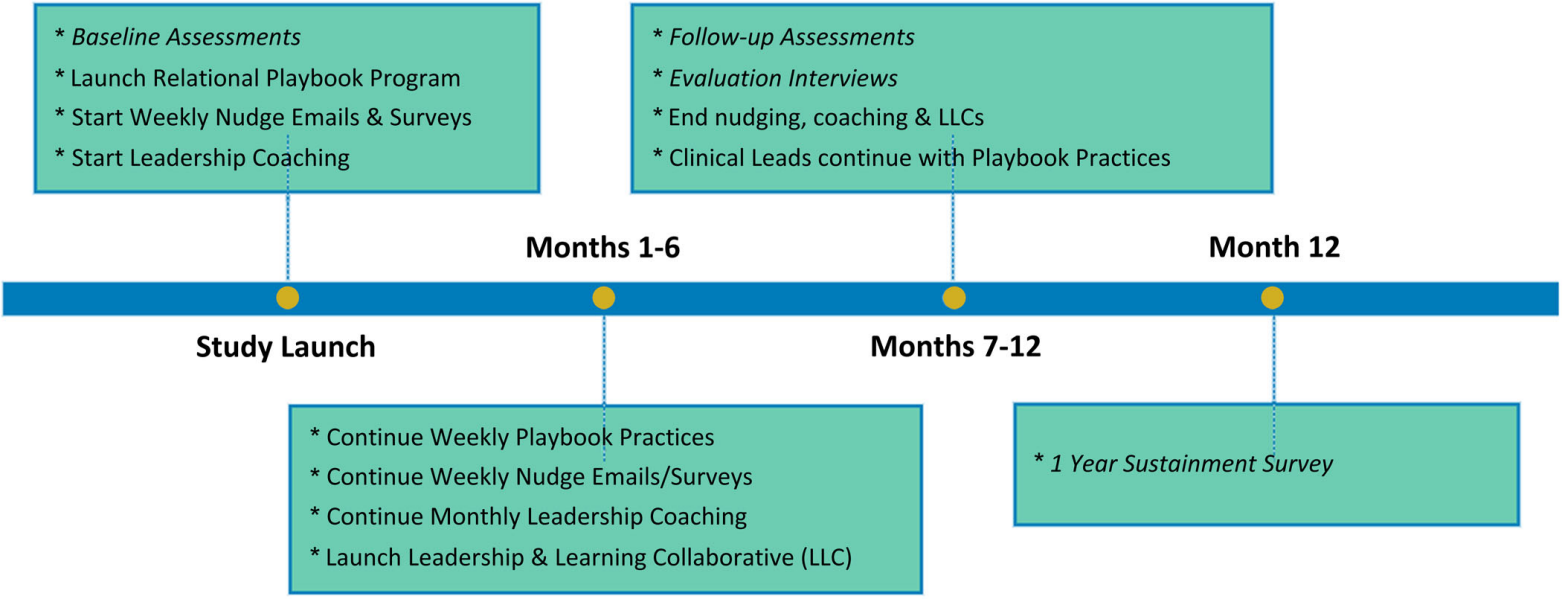
Acute Inpatient Medicine—High Reliability, Learning Environment, and Workforce Development InitiativeI implementation timeline.

This type II hybrid implementation study tests both the Playbook intervention and the implementation strategies.^18^ AIM‐HI implementation will occur in waves, enrolling additional sites every 12 months. Each implementation wave will follow three stages: preimplementation evaluation, implementation, and outcome evaluation. Preimplementation evaluation will assess facility context, staff and Veteran demographics, inpatient medical team composition, organizational culture, and the learning environment to tailor the Playbook intervention for each site. Six months after implementation, the formative evaluation will measure the initial intervention outcomes and provide performance feedback to each site. At 12 months after implementation, the summative evaluation will measure the overall success of the program outcomes and implementation strategies at each site and will be used to adapt future waves of implementation (Figure 2).

### Setting

In the first wave of implementation (2024), AIM‐HI will be implemented at three rural VA hospitals. Each site is from a different geographic VA region: New England, Southeast, and Southern regions. These are tertiary VA hospitals that treat at least 1000 rural Veterans annually, have an active inpatient hospital medicine program, and leadership support. Before the second wave of implementation, we will evaluate implementation of the first three sites to test the acceptability, appropriateness, and feasibility of the Playbook intervention and the impact of our implementation strategies. In subsequent waves, the Office of Rural Health will solicit for participation in AIM‐HI through a formal application process. Sites that serve large rural populations and have an active inpatient hospital medicine program will be encouraged to apply through targeted solicitation materials to hospital medicine leadership. Study participants will include inpatient physician, NP, PA, nursing, social work, pharmacy clinical leaders, and other clinical or leadership staff upon request.

### Description of implementation strategies

Based on previous experience working with rural facilities^19^ and pilot testing the Playbook in a VA cardiac catheterization laboratory, AIM‐HI will be supported by three evidence‐based implementation strategies: behavioral nudges, learning and leadership collaboratives, and leadership coaching. These strategies will be used during different phases of the initiative, guided by the Quality Enhancement Research Initiative Implementation Roadmap.^17^

#### Preimplementation data collection

##### Site visits

During the preimplementation phase, we will collect contextual data during a single‐day site visit conducted by three AIM‐HI team members. We will use a structured guide to observe clinical workflows and activities that support learning, high reliability, and workforce development activities. We will meet with clinical leaders to develop relationships, build a shared understanding of organizational goals, and how the AIM‐HI program can support these goals. Participants will be asked to report engagement with other VA workforce development programming (e.g., VA Employee Whole Health) during the study period.

##### Brainwriting premortem

Brainwriting premortem is a structured group exercise designed to identify contextual factors that may discourage implementation of an intervention.^20^ The method combines brainwriting, a collaborative written brainstorming technique, with a project premortem, where participants are asked to identify reasons a program will fail before implementation.^20^ The brainwriting premortem activity will be conducted at the end of each site visit to uncover hidden risks, engage AIM‐HI participants in proactive problem identification, and problem solving.

#### Implementation

AIM‐HI will use behavioral nudges, learning and leadership collaboratives, and leadership coaching as our evidence‐based implementation strategies to support Playbook intervention.

##### Behavioral nudge survey

To engage and activate clinical leaders implementing the Playbook we designed an email and corresponding Qualtrics survey using behavioral science principles.^21^ At the beginning of each week, the AIM‐HI team will send an email to notify clinical leaders of the Playbook learning and practices they will implement that week. The leaders will complete a brief, one question survey to set their weekly Playbook practice intention. At the end of the week, a five‐question Qualtrics survey will be sent to clinical leaders to facilitate reflection on the week's practices and collect standardized implementation, adoption, and fidelity data to inform formative and summative evaluations. The email and survey approaches are designed to remind clinical leaders to continue with Playbook implementation, encourage reflection, and facilitate real‐time data collection. Each site is expected to maintain fidelity to the Playbook interventions but will individualize the delivery of practices based on their local context. For instance, each site will be expected to implement gratitude practices with teams. However, the clinical leader can select the setting (e.g., team huddles, end of day debriefs) and timing (e.g., daily, weekly).

##### AIM‐HI learning and leadership collaborative

In the first month of implementation, the AIM‐HI team will host a launch meeting with each site and will be available to clinical leaders to identify barriers to intervention uptake and formulate solutions. In month 2, the AIM‐HI team will launch monthly 60‐min AIM‐HI learning and leadership collaborative meetings with participants at all sites. The meetings will provide brief education on Playbook practices, assist with implementation barriers and support, and build a community of practice between rural inpatient medicine teams to support sustained practice and growth. During these meetings sites will be invited to share stories and adaptations made to fit their local context.

##### Leadership coaching

In the first month of the Playbook implementation, clinical leaders implementing the Playbook practices will be offered executive leadership coaching to help them maximize their personal and professional performance as they change their culture toward learning and high reliability.^22^ The principal investigator for AIM‐HI (H. G.), an Associate Leadership Coach with the VA National Center for Organization Development (NCOD), will schedule an initial 1‐h coaching session followed by up to five 1‐h coaching sessions occurring every 2–4 weeks for 6 months on Microsoft Teams to all interested AIM‐HI participants. The initial coaching session will focus on creating a relationship, assessing needs, identifying values, setting goals, and creating a coaching plan. Subsequent sessions will follow the Grow, Reality, Options, Will framework,^22^ the guiding framework of NCOD coaching. In years 2 and 3 of AIM‐HI, we will continue to offer coaching and will recruit additional coaches through the VA NCOD Executive Leadership Coaching Cadre as needed.

#### Sustainment

AIM‐HI participants will be granted ongoing access to the Playbook when their sites move into the sustainment phase. The AIM‐HI team will be available to guide the ongoing use of the Playbook practices and will provide access to the Playbook for new staff from sites that have completed AIM‐HI, as requested. We will ask sites that have completed AIM‐HI to join monthly learning and leadership collaborative meetings in subsequent waves to share their experiences. The AIM‐HI team will create a manual that encapsulates lessons learned, common barriers to implementation, and solutions to these barriers to support the integration of the Playbook into VA National Hospital Medicine workforce development programming.

### AIM‐HI outcomes

We will use a mixed methods approach to evaluate implementation strategies and Playbook intervention outcomes across years and sites. The primary outcomes in year 1 will be the acceptability, appropriateness, and feasibility of AIM‐HI assessed through participant surveys and interviews. In subsequent years, trends in the learning environment and staff outcomes (e.g., job satisfaction, burnout, retention, turnover, and safety climate scores) will be evaluated. The AIM‐HI team will collect contextual data from existing VA data sources (e.g., VA Corporate Data Warehouse [CDW], VA All Employee Survey [AES], and 2022 VA Hospital Medicine Survey), AIM‐HI specific surveys, and site visit data, guided by the Reach, Effectiveness, Adoption, Implementation Framework (RE‐AIM).^23^

#### Implementation outcomes

Preimplementation data collection began in October 2024. During the preimplementation phase, we collect contextual data on workforce, veterans, and organizational characteristics. The preimplementation data are used to increase our understanding of the current composition of rural inpatient medical teams, the population they serve, and aspects of the organizational culture and the learning environment. The data also inform AIM‐HI implementation and adaptations, which will be tracked using a systematic, multimethod assessment of adaptations approach.^24^

Preimplementation data measures include facility and Veteran demographic data such as facility type, location, bed size, and Veteran services. Facility workforce data include hospital medicine team staffing structures and vacancy rates. Organizational culture will be assessed using VA AES results, a Google search using the terms “X VA news” to identify facility, staff or Veteran events reported in the lay media (e.g., awards, adverse events, public grievances), site visit observational notes, and brainwriting premortem results. Baseline organizational readiness for change is collected from site clinical leaders through a brief, theory‐based measure of organizational readiness to change.^25^ Baseline trends in the learning environment and staff outcomes (Table 1) are collected from clinical leaders and staff through the validated 64‐item Learning Environment Survey.^8^ Valid and reliable staff outcome measures were selected from the annual VA AES.

**Table 1:** Learning environment survey—staff outcome measures.

Measure	Survey item	Survey rating
Job satisfaction	Considering everything, how satisfied are you with your job?	Very Dissatisfied to Very Satisfied (5‐point Likert)
Burnout	I feel burned out from my work	Never to every day (7‐point Likert)
Turnover intention (retention)	If I were able, I would leave my current job	Strongly Disagree to Do Not Know (6‐point Likert)
Turnover	We have had ____ staff leave our team in the last 12 months	Choose one: 0; 1–2; 4–6; 7+
Safety climate	I would feel perfectly safe being treated in this department	Strongly Disagree to Do Not Know (6‐point Likert)

The preimplementation data will be analyzed by the AIM‐HI team, which includes a Veteran research partner, to inform the formative and summative evaluations. The quantitative and qualitative data will be triangulated and merged. To assess for multilevel contextual factors, the preimplementation data will be analyzed using PRISM.^11^

#### Intervention outcomes

The appropriateness, acceptability, and feasibility of the Playbook intervention will be assessed postimplementation using the valid and reliable implementation outcome measures developed by Weiner et al.^26^ The 12‐item survey queries the perception of participants using a 5‐point ascending Likert scale (completely disagree to completely agree). Acceptability is assessed by four questions, including “The Playbook meets my approval.” Appropriateness is assessed by four questions, including “The Playbook seems suitable.” Feasibility is assessed by four questions, including, “The Playbook seems easy to use.”^26^ The brief, easy to use measures were selected for they will provide actionable insights to inform Playbook adoption and adaptations. Postimplementation interviews will assess participant perceptions of the coaching, learning and leadership collaborative strategies, progress on quality improvement goals, and clinical leadership growth and development.

Additional RE‐AIM measures will include adoption/demand for AIM‐HI assessed through the number of hospital medicine clinical leaders engaged in AIM‐HI per site, the number and type of Playbook interventions implemented, and frequency and attendance at coaching, and learning and leadership collaborative sessions. Fidelity to the Playbook intervention components will be assessed through the weekly behavioral nudge survey data. Maintenance will be assessed by the number of Playbook interventions and attendance at learning and leadership collaborative sessions at 6 months and 1 year (Appendix A).

AIM‐HI ease of use, engagement, usefulness, adaptations, speed,^27^ cost of implementation, unintended consequences, and maintenance plans will be assessed through interviews with clinical leaders 6–9 months postimplementation. The interviews will be guided by the principles of Realist Evaluation,^28^ which seeks to understand what works, for whom, and under what circumstances. The interview guide includes questions that query the experience of learning about and participating in the Playbook, changes to inpatient medicine team workflow required to implement the Playbook, and organizational barriers and facilitators to effective implementation. To capture multiple perspectives, the AIM‐HI team will interview select clinical leaders and staff (*n* = 3–5) and a sample of hospital leaders who interact with inpatient medicine teams (*n* = 2–4) from each site. The interview guides will be pilot tested to ensure clarity, appropriateness, and relevance to the study questions. Interviews will last 30–45 min. Interviews will be audio recorded and transcribed verbatim.

The AIM‐HI team will use a SharePoint database for data auditing and reporting. Data analysis will be conducted in R software and *Atlas.ti* due to the mixed methods approach. The RE‐AIM evaluation results will be summarized and described. To characterize the cross‐sectional Learning Environment Survey and staff outcomes data, we will calculate descriptive, correlational, and reliability estimates. Changes in outcomes of interest from baseline to 6 months will be assessed using a linear mixed effect model with subject as a random effect and time as the predictor of primary interest.^29^ Interview data, coaching, and attendance records will be analyzed using an inductive‐deductive approach to qualitative content analysis.^29^ Deductive code categories will be used to categorize findings according to RE‐AIM and inductive codes will be added after discussion among coders to capture salient findings related to experience in and impact of the intervention, experience with coaching, and experience in the learning and leadership collaborative. Findings will be summarized by site, with attention to common themes across sites. The quantitative survey data will be triangulated with the qualitative data to assess for points of convergence and help explain how and why changes occurred. Integration of the mixed methods data will be achieved by reporting results together. The quantitative results will be reported first, followed by qualitative quotes or themes that support or refute the quantitative results.^30^

## ETHICS AND DISSEMINATION

AIM‐HI is an operationally funded, quality improvement program. The AIM‐HI protocol was approved by the VA Office of Rural Health and VA National Hospital Medicine Program. Participant consent is not required on the grounds that this is a minimal‐risk program. The AIM‐HI team will create a manual that encapsulates lessons learned, common barriers to implementation, and solutions to these barriers to support the integration of AIM‐HI into the VA Hospital Medicine workforce development programming. Dissemination of the study methods, tools, and implementation strategies will be through VA partners, national meetings, scientific journals, social media, and publications aimed at non‐VA settings.

## DISCUSSION

The anticipated impact of AIM‐HI is to evaluate the utility of the implementation strategies and assess trends in Playbook intervention outcomes, including enhanced job satisfaction, retention, safety climate, and reduced burnout in the rural inpatient setting. The Playbook has strong face validity; however, before large‐scale adoption across the VA enterprise, it is essential to establish the acceptability, appropriateness, and feasibility of the implementation strategies and the Playbook, as well as to gather data on its effectiveness. Rigorous implementation and evaluation within the rural hospital medicine team context will determine whether AIM‐HI is an appropriate hospital medicine workforce development program. Limitations of AIM‐HI include the voluntary nature of participation, which may result in selection bias towards participants more invested in personal leadership development. Challenges in recruiting participants and front‐line staff to complete surveys will be addressed by encouraging clinical leaders to allocate time during existing meetings for staff to complete the surveys.

## CONFLICT OF INTEREST STATEMENT

All authors receive some salary support from the Department of Veterans Affairs.

## ETHICS STATEMENT

The study was deemed an exempt human research study by the Colorado Multiple Institutional Review Board (COMIRB 17‐1153).

## APPENDIX A

Table A1

**Table A1:** AIM‐HI study measures and data collection timeline.

RE‐AIM measure	Outcomes	Time point (s)	Methods
**Reach**	N/A	N/A	N/A
**Effectiveness**	Acceptability, appropriateness, and feasibility of Playbook survey	6 months	Acceptability, appropriateness and feasibility survey
	Learning environment trends	Baseline and 6 months	Learning environment survey
	Staff outcome trends	Baseline and 6 months	Learning environment survey
	Progress on quality improvement goals	Baseline and 6 months	Learning environment survey
	Ease of use	6–9 months	Interviews
	Usefulness	6–9 months	Interviews; survey ratings on Playbook intervention, coaching intervention, and learning collaborative
	Engagement	6–9 months	Interviews; launch meeting, coaching and LLC attendance records
	Participant, site & veteran demographics Number of rural veterans served annually per site*	6–9 Baseline	CDW, AES, hospital medicine survey
**Adoption**	Number of inpatient medicine team members responding/receiving weekly surveys	Ongoing	Behavioral nudge survey
	Number/type Playbook interventions adopted	Ongoing	Behavioral nudge survey
	Attendance at coaching sessions	Ongoing	Attendance records
	Attendance at learning collaborative sessions	Ongoing	Attendance records
	Number of days from Playbook received to first trial	Baseline to first practice	Behavioral nudge survey
	Number of weeks for intervention to be integrated into practice	Baseline to first practice	Behavioral nudge survey
	Context for Implementation	Preimplementation; ongoing for each cohort	Analysis of hospital medicine survey. Site visits.
**Implementation**	Readiness to change	Baseline	Organizational readiness for implementing change survey
	Fidelity: Degree Playbook interventions delivered as intended	Ongoing	Behavioral nudge survey
	Adaptations to Playbook interventions	Ongoing, and at 6–9 months	Behavioral nudge survey, interviews; coaching records
	Cost (mean time/wage for participants, coach, and learning collaborative leaders)	6–9 months	Interviews; coaching records
	Unintended Consequences, adverse events, or concerns	6–9 months	LLC notes, interviews
	Number of Playbook interventions used at 1 year	12 months	Behavioral nudge survey
**Maintenance**	Attendance at learning collaborative sessions	1 year	Behavioral nudge survey, attendance records

*Note*: RE‐AIM measures noted in bold.

Abbreviations: AES, VA All Employee Survey; CDW, VA Corporate Data Warehouse; LLC, learning and leadership collaborative.
